# Reduced sphingolipid hydrolase activities, substrate accumulation and ganglioside decline in Parkinson’s disease

**DOI:** 10.1186/s13024-019-0339-z

**Published:** 2019-11-08

**Authors:** Mylene Huebecker, Elizabeth B. Moloney, Aarnoud C. van der Spoel, David A. Priestman, Ole Isacson, Penelope J. Hallett, Frances M. Platt

**Affiliations:** 1 Department of Pharmacology, University of Oxford, Oxford, OX1 3QT UK; 2 Neuroregeneration Institute, McLean Hospital / Harvard Medical School, Belmont, MA 02478 USA; 3 Departments of Pediatrics and Biochemistry & Molecular Biology, Atlantic Research Centre, Dalhousie University, Halifax, NS B3H 4R2 Canada

**Keywords:** Ageing, Glycosphingolipid, Ganglioside, Glucocerebrosidase, Lysosome, Neurodegeneration, Parkinson’s disease

## Abstract

**Background:**

Haploinsufficiency in the Gaucher disease *GBA* gene, which encodes the lysosomal glucocerebrosidase GBA, and ageing represent major risk factors for developing Parkinson’s disease (PD). Recently, more than fifty other lysosomal storage disorder gene variants have been identified in PD, implicating lysosomal dysfunction more broadly as a key risk factor for PD. Despite the evidence of multiple lysosomal genetic risks, it remains unclear how sphingolipid hydrolase activities, other than GBA, are altered with ageing or in PD. Moreover, it is not fully known if levels of glycosphingolipid substrates for these enzymes change in vulnerable brain regions of PD. Finally, little is known about the levels of complex gangliosides in substantia nigra which may play a significant role in ageing and PD.

**Methods:**

To study sphingolipid hydrolase activities and glycosphingolipid expression in ageing and in PD, two independent cohorts of human substantia nigra tissues were obtained. Fluorescent 4-methylumbelliferone assays were used to determine multiple enzyme activities. The lysosomal GBA and non-lysosomal GBA2 activities were distinguished using the inhibitor *N*B-DGJ. Sensitive and quantitative normal-phase HPLC was performed to study glycosphingolipid levels. In addition, glycosphingolipid levels in cerebrospinal fluid and serum were analysed as possible biomarkers for PD.

**Results:**

The present study demonstrates, in two independent cohorts of human post-mortem substantia nigra, that sporadic PD is associated with deficiencies in multiple lysosomal hydrolases (e.g. α-galactosidase and β-hexosaminidase), in addition to reduced GBA and GBA2 activities and concomitant glycosphingolipid substrate accumulation. Furthermore, the data show significant reductions in levels of complex gangliosides (e.g. GM1a) in substantia nigra, CSF and serum in ageing, PD, and REM sleep behaviour disorder, which is a strong predictor of PD.

**Conclusions:**

These findings conclusively demonstrate reductions in GBA activity in the parkinsonian midbrain, and for the first time, reductions in the activity of several other sphingolipid hydrolases. Furthermore, significant reductions were seen in complex gangliosides in PD and ageing. The diminished activities of these lysosomal hydrolases, the glycosphingolipid substrate accumulation, and the reduced levels of complex gangliosides are likely major contributors to the primary development of the pathology seen in PD and related disorders with age.

## Background

Parkinson’s disease (PD) is the second most common, late-onset neurodegenerative disease after Alzheimer’s disease and is characterised by the degeneration of dopaminergic neurons within the substantia nigra (SN). Loss of dopaminergic neurons results in disrupted motor control, causing tremor, rigidity, bradykinesia and gait dysfunction [1]. Only 5–10% of PD cases have been linked to a genetic cause, whilst 90% of PD cases are sporadic [2]. Ageing is the greatest non-genetic risk factor for PD, with most PD patients being over the age of 60 [1]. The brain is especially vulnerable to progressive age-related changes, as alterations in multiple biological pathways may impair dopaminergic and other vulnerable neurons, and therefore lower the threshold for developing PD [3].

Mutations in lysosomal genes are the cause of more than 70 rare lysosomal storage diseases (LSDs), which often have a relentless neurodegenerative clinical course [4, 5]. Gaucher disease (GD) is one of the most common autosomal LSDs and is caused by mutations in *GBA*, which encodes the lysosomal glucocerebrosidase GBA. A significant reduction in GBA activity results in accumulation of its glycosphingolipid (GSL) substrates, glucosylceramide (GlcCer) and glucosylsphingosine (GlcSph). Heterozygote mutations in *GBA* significantly increase the risk of PD [6–10]. Reduced GBA activity has been reported in brain tissue from both PD-GBA patients and sporadic PD patients without GBA mutations, suggesting a broader role for the lysosome in PD [11–13]. Furthermore, GBA activity has been shown to decline progressively with ageing in the SN and putamen of healthy controls, eventually becoming comparable to GBA activity found in PD patients [12].

The activity of GBA needs to be reliably distinguished from that of β-glucosidase 2 (GBA2), which is a non-lysosomal enzyme that cleaves the same natural and artificial substrates as GBA [14–17]. GBA2 has been reported to be localised at the plasma membrane [15, 18, 19] and cytoplasmic face of the endoplasmatic reticulum and Golgi [20], and is highly expressed in testis, liver, and brain, in particular in Purkinje cells [14, 16, 21]. Pharmacological inhibition of GBA2 in mice results in higher levels of GlcCer in testis, brain, and liver [22–24]; ablation of the *GBA2* gene has similar consequences [16, 25]. In humans, mutations in the *GBA2* gene result in neurological conditions on the ataxia-spasticity spectrum [26–28].

GBA has been shown to interact with α-synuclein [29, 30], providing a plausible biological relationship between GD and PD. Furthermore, GlcCer, the substrate for GBA, was shown to directly stabilise oligomeric intermediates of α-synuclein in a lysosome-like environment, which led to further depletion of lysosomal GBA activity, generating a self-propagating positive feedback loop culminating in neurodegeneration [31]. Further studies have supported a link between the lipids GlcCer and GlcSph with α-synuclein [32–34]. For example, both GlcSph and GlcCer have been reported to promote the formation of oligomeric α-synuclein species in GBA-associated PD [32, 33]. iPSC-derived dopaminergic neurons from GBA-associated PD patients also exhibited elevated levels of GlcCer [35]. It has also been shown that lipid changes occur in sporadic PD patients [12, 36]. However, other reports have stated that levels of GlcCer and GlcSph do not increase in either GBA-associated PD or sporadic PD [37, 38]. Dysregulation of GSLs in PD was further implicated in studies of mice lacking major brain gangliosides, in particular GM1a, which were found to develop Parkinsonism [39]. Gangliosides are complex GSLs, which are the most abundant GSLs in the CNS in all mammals and essential for brain function [40, 41]. Ganglioside GM1a is essential for myelination, neuritogenesis, synaptogenesis and signalling of the neurotrophic factor GDNF [42–44]. A reduction in GM1a levels was described in SN and occipital cortex from PD patients [42, 45, 46].

Besides the link between GBA/GD and PD, there have been several reports linking other LSDs, e.g. Fabry disease and Niemann Pick type C disease, with PD [47–51]. Recently, an excessive burden of LSD gene variants was found in PD patients, consistent with lysosomal dysfunction representing a risk factor for PD [52].

Here, we therefore investigated whether PD- and ageing-induced changes in brain GSL homeostasis and lysosomal hydrolase activities occur more broadly in tissues from control subjects and PD patients. In addition to shedding light on pathogenesis of PD, these studies have also identified potential novel lipid-related biomarkers.

## Materials and methods

### Patients

Frozen post-mortem substantia nigra (SN) from neurologically unaffected patients (healthy control subjects) and sporadic PD patients was provided by the Harvard Brain Tissue Resource Centre (HBTRC; McLean Hospital, Belmont, MA) and the Parkinson’s UK Brain Bank (PDUK; Imperial College London, UK). All PD cases met a pathological diagnosis of PD made by the brain banks, which was based on the extent of neuronal (pigment) loss in the SN and Braak staging. Available clinical data, e.g. α-synuclein and Tau Braak scores, are summarised in Tables 1, 2, 3. Data of individual patients can be found in Additional file 2.

**Table 1:** Parkinson’s disease and control case information from substantia nigra received from HBTRC

	Control subjects (70s cohort)	Control subjects (80s cohort)	PD subjects (70s cohort)	PD subjects (80s cohort)
Cohort size	10	10	10	8
Female (%)	20.0	50.0	10.0	37.5
Male (%)	80.0	50.0	90.0	62.5
Age (years)	71.2 ± 2.9	81.6 ± 4.8	71.0 ± 3.3	81.9 ± 3.9
PMI (hours)	23.0 ± 4.6	22.7 ± 6.5	16.3 ± 6.4	17.1 ± 6.0
Disease duration (years)	N/A	N/A	15.3 ± 8.2^a^	17.4 ± 8.4^a^
α-synuclein Braak score	N/A	N/A	Score 4: *n* = 5 Score 5: *n* = 5	Score 1: *n* = 1 Score 3: *n* = 1 Score 4: *n* = 3 Score 5: *n* = 3
Tau Braak score	N/A	N/A	Score 1 *n* = 2 Score 2: *n* = 5 Score 3: *n* = 3	Score 1 *n* = 2 Score 2: *n* = 5 Score 3: *n* = 1
Neuronal loss in SN	Score 0: *n* = 10	Score 0: *n* = 8 Score 0.5: *n* = 2	Score 2: *n* = 1 Score 3: *n* = 9	Score 1: *n* = 1 Score 2.5: *n* = 1 Score 3: *n* = 6

*PMI* post-mortem interval. Data summarised as mean ± SD. α-synuclein Braak score (ranging from 1 to 6) based on Braak et al., 2003, Neurobiol Aging. Tau pathology Braak score (ranging from 1 to 6) based on Braak et al., 1991, Acta. Neuropathologica. Score for neuronal loss is semi-quantitative with 0 = no loss and 4 = extreme loss. ^a^Disease duration not available for 3 cases in 70s PD cohort, and 1 case in 80s PD cohort

1. Braak H, Del Tredici K, Rub U, de Vos RA, Jansen Steur EN, Braak E. Staging of brain pathology related to sporadic Parkinson’s disease. Neurobiol Aging. 2003;24(2):197–211

2. Braak H, Braak E. Neuropathological stageing of Alzheimer-related changes. Acta Neuropathol. 1991;82(4):239–59

**Table 2:** Parkinson’s disease and control case information from substantia nigra received from PDUK Brain Bank

	Control subjects	PD subjects
Cohort size	5	20
Female (%)	20	20
Male (%)	80	80
Age (years)	83.4 ± 9.5	77.6 ± 6.9
PMI (hours)	15.4 ± 6.9	15.9 ± 5.2
Disease duration (years)	N/A	13.5 ± 7.4
α-synuclein Braak score	N/A	Score 5: *n* = 5 Score 6: *n* = 15
Tau Braak score	N/A	Score 1 *n* = 8 Score 2: *n* = 11 Score 3: *n* = 1

*PMI* post-mortem interval. Data summarised as mean ± SD. α-synuclein Braak score (ranging from 1 to 6) based on Alafuzoff et al., 2009, Acta Neuropathologica. Tau pathology Braak score (ranging from 1 to 6) based on Alafuzoff et al., 2008, Brain Pathology

3. Alafuzoff I, Ince PG, Arzberger T, Al-Sarraj S, Bell J, Bodi I, et al. Staging/typing of Lewy body related alpha-synuclein pathology: a study of the BrainNet Europe Consortium. Acta Neuropathol. 2009;117 (6):635–52

4. Alafuzoff I, Arzberger T, Al-Sarraj S, Bodi I, Bogdanovic N, Braak H, et al. Staging of neurofibrillary pathology in Alzheimer’s disease: a study of the BrainNet Europe Consortium. Brain Pathol. 2008;18 (4):484–96

**Table 3:** Parkinson’s disease, RBD and control case information from ante-mortem CSF and serum received from OPDC

	Control subjects	PD subjects	RBD subjects
Cohort size	15	30	30
Female (%)	53.3	50.0	13.3
Male (%)	46.7	50.0	86.7
Age (years)	66.2 ± 8.4	63.8 ± 9.9	64.4 ± 11.6
H&Y score	N/A	Score 1: *n* = 6 Score 2: *n* = 21 Score 3: *n* = 3	N/A

*RBD* REM sleep behaviour disorder. Only serum was available for RBD cases. Data summarised as mean ± SD

From the HBTRC, post-mortem SN tissue from healthy subject controls (*n* = 20) and PD patients (*n* = 18), which were closely matched for age, sex, and post-mortem interval, were provided (Table 1). The PD patient brain tissue was sequenced for GBA mutations (the GBA pseudogene was also taken into account) at Beckman Coulter Genomics (Danvers, MA). Four PD patients were found to be GBA mutation carriers: AN14826 (L444P), AN01359 (V294 M), AN10183 (E326K) and AN07327 (E326K). PD patients with GBA mutation were not removed from further analysis as no statistically significant differences were observed to sporadic PD cases. However, for the reader’s convenience, PD patients, who were identified as GBA mutation carriers, are coloured in grey, to be distinguishable from sporadic PD patients coloured in black. The PDUK brain bank provided a second, independent cohort of post-mortem SN tissue from healthy control subjects (*n* = 5) and age-matched PD patients (*n* = 20) (Table 2). Tissues were rapidly homogenised in PBS using a handheld Ultraturax T25 probe homogeniser (IKA, Germany) and aliquoted before being stored at − 80 °C.

Furthermore, for biomarker studies, frozen ante-mortem cerebrospinal fluid (CSF) and serum samples from control subjects and PD patients were provided by the Oxford Parkinson’s Disease Centre (OPDC; Oxford, UK). Ante-mortem CSF of control subjects (*n* = 15, mean age: 66 years) and age-matched PD subjects (*n* = 28, mean age: 64 years) was used for GSL analysis (Table 3). Furthermore, serum samples from patients at risk of developing PD (prodromal PD phase), diagnosed with rapid eye movement (REM) sleep behaviour disorder (RBD), were provided. RBD is a parasomnia which involves acting out dreams and abnormal movements during REM sleep stage. RBD patients have an 80–90% risk of conversion to a synucleinopathy disorder (e.g. PD or dementia with Lewy bodies) over 14 years from the time of RBD diagnosis [53–55]. Serum of control subjects (*n* = 15, mean age: 66 years), PD patients (*n* = 30, mean age: 64 years) and RBD patients (*n* = 30, mean age: 64 years) were used for GSL analysis (Table 3).

### Lysosomal hydrolase activity assays

Lysosomal hydrolase activities were assayed fluorometrically using artificial sugar-substrates conjugated with the fluorophore 4-methylumbelliferone (4-MU). For measuring β-glucosidase activities, samples were incubated in the presence or absence of 0.3 mM *N*B-DGJ for 30 min on ice prior to the assay. The substrate for GBA β-glucosidase activity was 4.5 mM 4-MU β-D-glucoside in 200 mM citrate/phosphate buffer, pH 5.2, 0.25% TritonX-100, 0.25% sodium taurocholate, 1.25 mM EDTA and 4 mM 2-mercaptoethanol. GBA activity was defined as the *N*B-DGJ non-sensitive activity at pH 5.2. The substrate for GBA2 β-glucosidase activity was 4.5 mM 4-MU β-D-glucoside in 200 mM citrate/phosphate buffer, pH 5.5, 0.1% TritonX-100. GBA2 activity was defined as the *N*B-DGJ sensitive activity at pH 5.5. For α-galactosidase activity, 5 mM 4-MU α-D-galactoside in 100 mM sodium citrate buffer, pH 4.0, 0.1% TritonX-100 was used as substrate. For β-hexosaminidase activity, 3 mM 4-MU N-acetyl-β-D-glucosaminide in 200 mM sodium citrate buffer, pH 4.5, 0.1% TritonX-100 was used as substrate. For β-galactosidase activity, 1 mM 4-MU β-D-galactopyranoside in 200 mM sodium acetate buffer, pH 4.3, 100 mM NaCl, 0.1% TritonX-100 was used as substrate. The substrate for neuraminidase activity was 0.8 mM 4-MU N-acetylneuraminic acid in 0.1 M acetate buffer, pH 4.6, 0.1% TritonX-100. The digests (in triplicate) containing tissue homogenate in PBS with 0.1% TritonX-100 and artificial 4-MU substrate were incubated at 37 °C for 30 min (or 2 h for neuraminidases). The reaction was stopped by adding cold 0.5 M Na_2_CO_3_ (pH 10.7). The released fluorescent 4-MU was measured in a FLUOstar OPTIMA plate reader (BMG Labtech, Ortenberg, Germany) with an excitation at 360 nm and emission at 460 nm. A standard curve of free 4-MU was used to calculate the enzyme activity. Results were normalised to protein content.

### GlcCer and GSL analysis with NP-HPLC

GlcCer and downstream GSLs were analysed essentially as described by Neville and co-workers [56]. Lipids from tissue homogenates or body fluids were extracted with chloroform and methanol overnight at 4 °C. The GSLs were then further purified using solid-phase C18 columns (Telos, Kinesis, UK). After elution, the GSL fractions were split in half, dried down under a stream of nitrogen at 42 °C and treated with either Cerezyme® (Genzyme, Cambridge, MA) to obtain glucose from GlcCer or recombinant ceramide glycanase (rEGCase, prepared by Genscript and provided by Orphazyme, Denmark) to obtain oligosaccharides from more complex GSLs. The liberated glucose and free glycans were then fluorescently-labelled with anthranillic acid (2AA). To remove excess 2AA label, labelled glycans were purified using DPA-6S SPE columns (Supelco, PA, USA). Purified 2AA-labelled glucose and 2AA-labelled oligosaccharides were separated and quantified by normal-phase high-performance liquid chromatography (NP-HPLC) as previously described [56]. The NP-HPLC system consisted of a Waters Alliance 2695 separations module and an in-line Waters 2475 multi λ-fluorescence detector set at Ex λ360 nm and Em λ425 nm. The solid phase used was a 4.6 × 250 mm TSK gel-Amide 80 column (Anachem, Luton, UK). A 2AA-labelled glucose homopolymer ladder (Ludger, UK) was included to determine the glucose unit values (GUs) for the HPLC peaks. Individual GSL species were identified by their GU values and quantified by comparison of integrated peak areas with a known amount of 2AA-labelled BioQuant chitotriose standard (Ludger, UK). Results for tissue homogenates were normalised to protein content, determined by the bicinchoninic acid (BCA) assay.

### Sphingosine and GlcSph analysis with RP-HPLC

Sphingosine, sphinganine and glucosylsphingosine from substantia nigra homogenates were extracted in chloroform:methanol (1:2, v/v) with sonication for 10 min at room temperature. Lipids were purified using SPE NH2 columns (Biotage, #470–0010-A). After elution, sphingosine species were labelled with o-Phthalaldehyde (OPA) for 20 min at room temperature in the dark and OPA-labelled lipids were taken for analysis by reverse phase high-performance liquid chromatography (RP-HPLC). The RP-HPLC system consisted of a VWR Hitachi Elite LaChrom HPLC system with a L-2485 fluorescence detector set at Ex λ340nm and Em λ455nm. The solid phase used was a Chromolith Performance RP-18e 100–4.6 HPLC column (Merck, Darmstadt, Germany). Individual sphingosine species were identified by their retention time and quantified by comparison of integrated peak areas with a known amount of OPA-labelled C20 sphingosine standard (Avanti Polar Lipids, Alabama, USA) or OPA-labelled C20 glucosylsphingosine standard (Avanti Polar Lipids, Alabama, USA), respectively. Results were normalised to protein content.

### Cholesterol quantification

Total cholesterol (free cholesterol and cholesteryl esters) was quantified using the Amplex Red Cholesterol Assay Kit (Thermo Fisher Scientific, UK), according to manufacturer’s instructions. Results were normalised to protein content.

### Statistical analysis

All statistical analyses were performed with GraphPad Prism 7.0 (GraphPad, San Diego, CA). Unpaired student’s *t*-test was used to compare two groups and one-way or two-way ANOVA followed by post-hoc tests (as appropriate) was used to compare multiple groups. Correlations were analysed with Pearson correlation analysis.

## Results

### GBA and GBA2 activities progressively decline in the substantia nigra with normal ageing and are further decreased in PD

To investigate if activities of the β-glucosidases GBA and GBA2 are altered in ageing or PD, GBA and GBA2 activities were assayed in substantia nigra (SN) from healthy control subjects and PD patients. Patients and controls were divided into those in their 7th or 8th decades of life, termed here the 70s-cohort and 80s-cohort (*n* = 10 per condition and *n* = 5 per age group, provided by Harvard Brain Tissue Resource Centre (HBTRC)). PD patients, which were identified as GBA mutation carriers (see Materials and Methods for details), are coloured in grey, to be distinguishable from sporadic PD patients coloured in black.

**Fig. 1:**
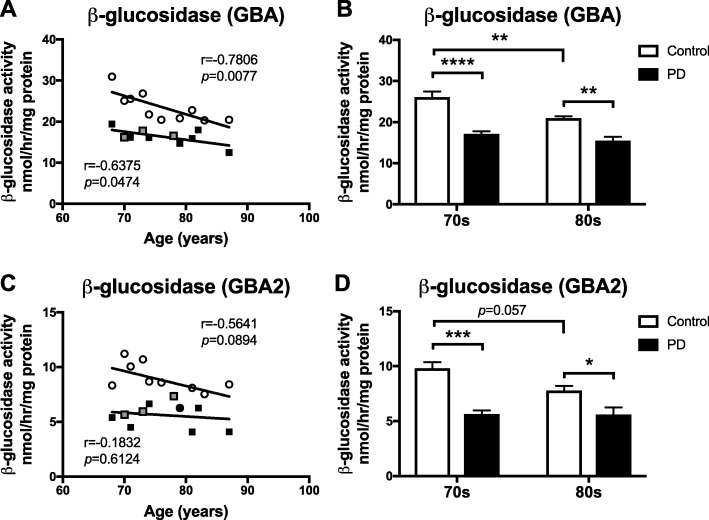
Reduced GBA and GBA2 activities in substantia nigra of PD patients and with normal ageing. GBA and GBA2 β-glucosidase activities were measured using artificial 4-MU-substrate and the inhibitor *N*B-DGJ. Activity of GBA (**a**,**b**) and GBA2 (**c**,**d**) were determined in substantia nigra from control subjects and PD patients. PD patients identified as GBA mutation carriers are shown in grey (a,c). Data were analysed using Pearson correlation analysis (**a**,**c**) (*n* = 10 per group) and 2-way ANOVA (**b**,**d**) (*n* = 5 per cohort; * = *p* < 0.05, ** = *p* < 0.01, *** = *p* < 0.001, **** = *p* < 0.0001). All *p*-values can be found in the Additional file 3. Bar graphs are presented as mean ± SEM

GBA activity in substantia nigra was negatively correlated with the age of control subjects and PD patients (Fig. 1a). GBA activity in the substantia nigra of 80s-cohort control subjects was significantly reduced to 80.5% of the GBA activity in the control 70s-cohort (Fig. 1b). There was no difference in SN GBA activity between the 70s-cohort and 80s-cohort of PD patients (Fig. 1b). The activity of GBA in SN of PD patients was significantly reduced by 34.2% in the 70s-cohort and 26.0% in the 80s-cohort compared to age-matched controls (Fig. 1b).

GBA2 activity in substantia nigra had a mild trend towards a negative correlation with the age of control subjects, but not with the age of PD patients (Fig. 1c). There was a trend towards a reduction in GBA2 activity in SN of 80s-cohort control subjects compared to 70s-cohort control subjects (20.6% reduction, Fig. 1d). No difference in SN GBA2 activity between the 70s-cohort and 80s-cohort of PD patients was found (Fig. 1d). A significant decrease in GBA2 activity was seen in SN of 70s-cohort PD patients (42.6% reduction) and 80s-cohort PD patients (27.9% reduction) compared to the corresponding age-matched controls (Fig. 1d).

### Reduced activity of lysosomal hydrolases in substantia nigra of PD patients

As mutations in multiple lysosomal hydrolases were recently identified as potential risk factors for PD, additional lysosomal enzymes were assayed in substantia nigra from control subjects and PD patients (*n* = 10 per condition and *n* = 5 per age group, provided by HBTRC).

**Fig. 2:**
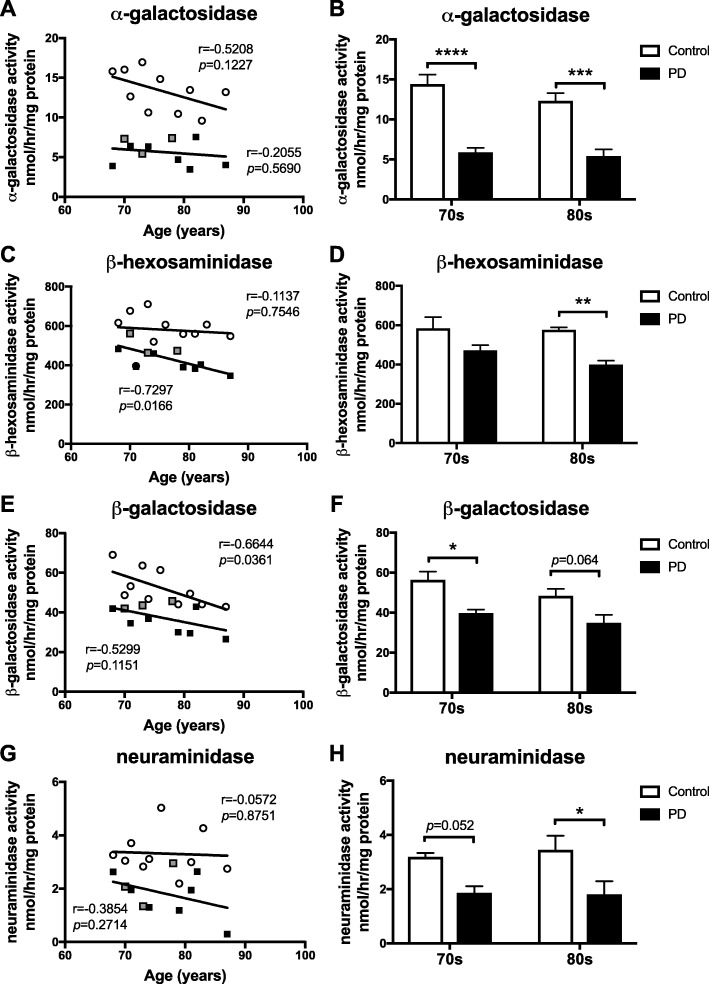
Reduced lysosomal hydrolase activities in substantia nigra of PD patients. Lysosomal hydrolase activities were measured using artificial 4-MU-substrates. Activity of α-galactosidase (**a**,**b**), β-hexosaminidase (**c**,**d**), β-galactosidase (**e**,**f**) and neuraminidase (**g**,**h**) were determined in substantia nigra from control subjects and PD patients. PD patients identified as GBA mutation carriers are shown in grey (**a**,**c**,**e**,**g**). Data were analysed using Pearson correlation analysis (**a**,**c**,**e**,**g**) (*n* = 10 per group) and 2-way ANOVA (**b**,**d**,**f**,**h**) (*n* = 5 per cohort; * = *p* < 0.05, ** = *p* < 0.01, *** = *p* < 0.001, **** = *p* < 0.0001). All *p*-values can be found in the Additional file 3. Bar graphs are presented as mean ± SEM

Substantia nigra α-galactosidase activity had a trend towards a negative correlation with increasing age in control subjects, but less so in PD patients (Fig. 2a). A significant reduction in α-galactosidase activity was however observed in SN of 70s-cohort PD patients (59.2% reduction) and 80s-cohort PD patients (55.9% reduction) compared to age-matched controls (Fig. 2b). No change in Gb3, the principle GSL substrate for α-galactosidase, levels in the substantia nigra of PD patients compared to control subjects was observed (Additional file 1: Figure S1A, B).

β-hexosaminidase activity was significantly, negatively correlated with the age of PD patients, but not in control subjects (Fig. 2c). A significant reduction in β-hexosaminidase activity was observed in the 80s-cohort PD patients compared to 80s-cohort control subjects (30.7% reduction, Fig. 2d).

Substantia nigra β-galactosidase activity was significantly, negatively correlated with the age of control subjects, and had a negative correlation with the age of PD patients (Fig. 2e). The activity of β-galactosidase in substantia nigra of 70s-cohort PD patients was significantly reduced to 70.7% of β-galactosidase activity of age-matched control subjects (Fig. 2f). There was a trend to reduced activity in PD patients when comparing β-galactosidase activities of both 80s-cohorts (27.8% reduction).

Neuraminidase activity in substantia nigra of control subjects and PD patients was not significantly correlated with age (Fig. 2g). However, decreased neuraminidase activity was observed in SN of 70s-cohort PD patients compared to 70s-cohort control subjects (41.7% reduction, Fig. 2h), but did not reach statistical significance. Activity of neuraminidase in substantia nigra of the 80s-cohort PD patients was significantly reduced to 52.4% of the activity in SN of age-matched control subjects (Fig. 2h).

### Accumulation of glucosylceramide and glucosylsphingosine in substantia nigra of PD patients

Levels of glucosylceramide (GlcCer), one of the substrates for GBA and GBA2, and lactosylceramide (LacCer), sequential precursors of all more complex GSLs in the biosynthetic pathway, were quantified in substantia nigra from PD patients (*n* = 18) and age-matched controls (*n* = 20, provided by HBTRC) by NP-HPLC.

**Fig. 3:**
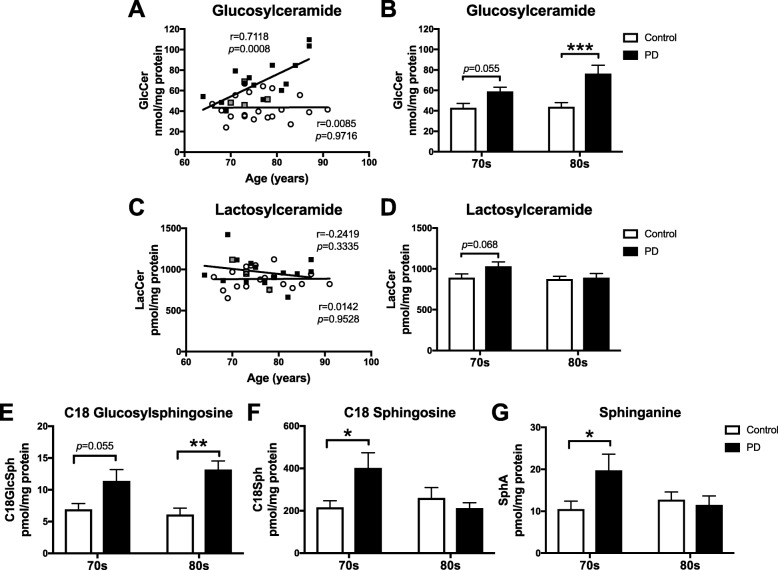
Glucosylceramide and glucosylsphingosine levels are increased in the substantia nigra of PD patients. **a**-**d** Substantia nigra from control subjects (*n* = 20) and PD patients (*n* = 18) were used to determine glucosylceramide (GlcCer) and lactosylceramide (LacCer) levels with NP-HPLC. **a**, **c** Data were analysed using Pearson correlation analysis. **b**, **d** Comparison of GlcCer and LacCer levels in 70s-cohorts and 80s-cohorts of control subjects and PD patients was performed using 2-way ANOVA (*n* = 8–10 per cohort, *** = *p* < 0.001). PD patients identified as GBA mutation carriers are shown in grey (**a**,**c**). **e**-**g** Substantia nigra from control subjects and PD patients were used to determine glucosylsphingosine (GlcSph), sphingosine (Sph) and sphinganine (SphA) levels with RP-HPLC. **e** GlcSph levels in 70s-cohorts and 80s-cohorts of control subjects and PD patients (*n* = 5 per cohort, ** = *p* < 0.01, 2-way ANOVA). **f** Sph levels in 70s-cohorts and 80s-cohorts of control subjects and PD patients (*n* = 5 per cohort, * = *p* < 0.05, 2-way ANOVA). **g** SphA levels in 70s-cohorts and 80s-cohorts of control subjects and PD patients (*n* = 5 per cohort, * = *p* < 0.05, 2-way ANOVA). Bar graphs are presented as mean ± SEM

GlcCer levels were significantly, positively correlated with increasing age in substantia nigra of PD patients, but not in control subjects (Fig. 3a). In the 70s-cohort PD patients, GlcCer levels in substantia nigra were increased to 137.1% of age-matched control subjects, but did not reach statistical significance (Fig. 3b). In substantia nigra of 80s-cohort PD patients, GlcCer levels were significantly increased to 174.0% of 80s-cohort control subjects (Fig. 3b). Exemplary NP-HPLC traces of GlcCer extracted from substantia nigra of 80s-cohort control subjects and PD patients are shown in Additional file 1: Figure S2A.

For LacCer, there was no significant correlation with age in substantia nigra of control subjects and PD patients (Fig. 3c). There were also no significant changes observed when comparing substantia nigra LacCer levels between control and PD cohorts at different ages (Fig. 3d).

Levels of glucosylsphingosine (GlcSph), another substrate for GBA and GBA2, as well as levels of sphingosine (Sph) and sphinganine (SphA) were quantified in substantia nigra of PD patients and age-matched controls (*n* = 10 per condition and *n* = 5 per age group) using RP-HPLC. In 70s-cohort PD patients, GlcSph levels in substantia nigra were increased to 164.4% of age-matched control subjects, but did not reach statistical significance (Fig. 3e). In substantia nigra of 80s-cohort PD patients, GlcSph levels were significantly increased to 215.9% of the 80s-cohort control subjects (Fig. 3e). Both sphingosine and sphinganine levels were significantly increased in substantia nigra of 70s-cohort PD patients in comparison to age-matched control subjects (Sph: 86.2% increase; SphA: 87.5% increase, Figs. 3f, g).

Cholesterol levels were measured in substantia nigra tissues of PD patients and age-matched controls (*n* = 10 per condition and *n* = 5 per age group) using Amplex Red assay. No differences in cholesterol levels were seen (Additional file 1: Figure S3).

### Loss of gangliosides GM1a, GD1a, GD1b and GT1b in substantia nigra with normal ageing and further in PD

**Fig. 4:**
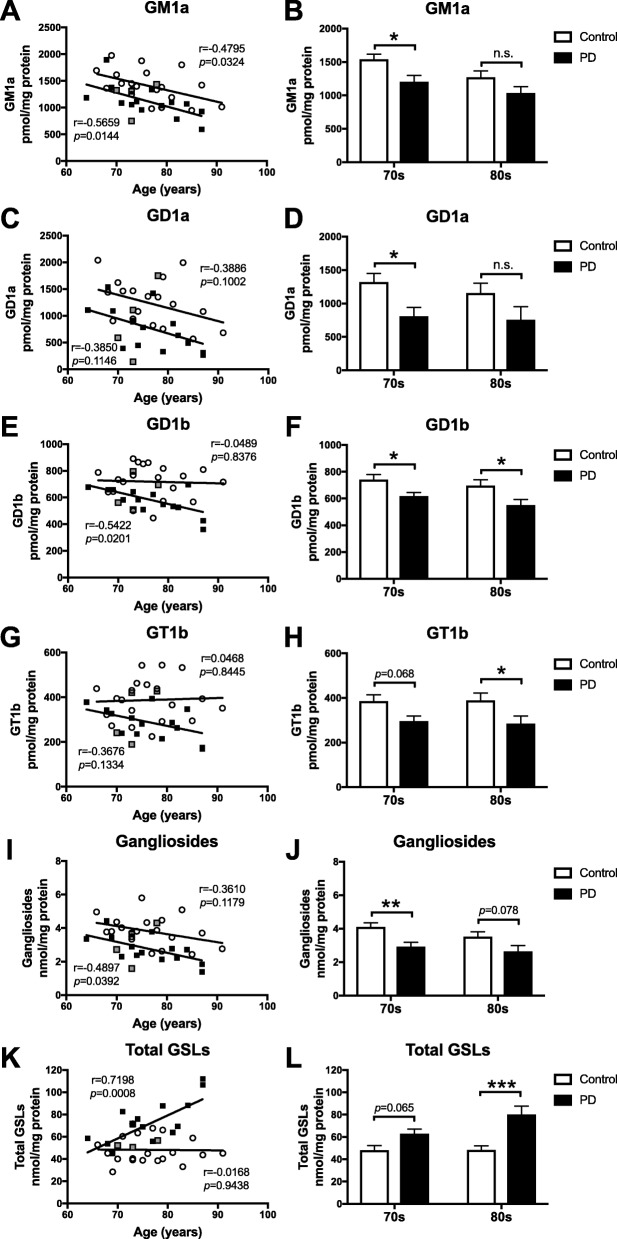
Loss of gangliosides in substantia nigra of PD patients and with normal ageing. Levels of GM1a (**a**,**b**), GD1a (**c**,**d**), GD1b (**e**,**f**) and GT1b (**g,h**) were determined in substantia nigra from control subjects and PD patients with NP-HPLC. Data were analysed using Pearson correlation analysis (**a**,**c**,**e**,**g**) (*n* = 18–20 per group) and 2-way ANOVA (**b**,**d**,**f**,**h**) (*n* = 8–10 per cohort; * = *p* < 0.05). **i** Pearson correlation analysis of the sum of GM1a + GD1a + GD1b + GT1b levels in substantia nigra from control subjects (*n* = 20) and PD patients (*n* = 18). **j** Comparison of ganglioside levels in 70s-cohort vs. 80s-cohort of control subjects and PD patients (*n* = 8–10 per cohort, ** = *p* < 0.01, 2-way ANOVA). **k** Pearson correlation analysis of the sum of GlcCer + LacCer + GM1a + GD1a + GD1b + GT1b levels in substantia nigra from control subjects (*n* = 20) and PD patients (*n* = 18) shows that PD is associated with increased GSL load with age. **i** Comparison of total GSL levels in 70s-cohorts vs. 80s-cohorts of control subjects and PD patients (*n* = 8–10 per cohort, *** = *p* < 0.001, 2-way ANOVA). PD patients identified as GBA mutation carriers are shown in grey (**a**,**c**,**e**,**g**,**i**,**k**). Bar graphs are presented as mean ± SEM

Levels of the more-complex gangliosides, GM1a, GD1a, GD1b and GT1b, were quantified in substantia nigra from PD patients (*n* = 18) and age-matched controls (*n* = 20) by NP-HPLC (provided by HBTRC). GM1a levels were significantly, negatively correlated with increasing age in substantia nigra of both control subjects and PD patients (Fig. 4a). A significant decrease in GM1a levels was observed in SN of 70s-cohort PD patients compared to age-matched control subjects (21.8% reduction, Fig. 4b), but was not significant when comparing 80s-cohorts. A negative correlation with age in GD1a substantia nigra levels of control subjects and PD patients was found (Fig. 4c). A significant decrease in GD1a levels was also observed in SN of 70s-cohort PD patients compared to 70s-cohort control subjects (38.7% reduction, Fig. 4d). GD1b and GT1b levels in substantia nigra of PD patients were both negatively correlated with age, but not in substantia nigra of control subjects (Fig. 4e, g). GD1b levels in substantia nigra of PD patients of both age cohorts were significantly reduced compared to substantia nigra of age-matched control subjects (70s: 16.5% reduction; 80s: 21.0% reduction, Fig. 4f). Similarly, a decrease in GT1b levels in substantia nigra of PD patients in both age cohorts was observed relative to age-matched controls (70s: 23.3% reduction; 80s: 26.9% reduction, Fig. 4h). Exemplary NP-HPLC traces of GM1a, GD1a, GD1b and GT1b from substantia nigra of 80s-cohort control subjects and PD patients are shown in Additional file 1: Figure S2B. We calculated total levels of more-complex gangliosides by summing GM1a, GD1a, GD1b and GT1b. Ganglioside levels in substantia nigra of PD patients were significantly negatively correlated with age, and also negatively correlated in substantia nigra of control subjects (Fig. 4i). In substantia nigra of 70s-cohort PD patients, ganglioside levels were significantly decreased to 71.3% of age-matched control subjects (Fig. 4j). In substantia nigra of 80s-cohort PD patients, ganglioside levels were decreased to 75.0% of 80s-cohort control subjects, trending towards statistical significance (Fig. 4j). To assess whether total brain glycosphingolipid (GSL) load in substantia nigra changes with healthy ageing or with PD, GlcCer, LacCer and ganglioside levels were summed and termed total GSLs. The total amount of GSLs is mostly influenced by the highly abundant GlcCer. Total GSL levels in substantia nigra of PD patients were significantly, positively correlated with age, but not in substantia nigra of control subjects (Fig. 4k). In 70s-cohort PD patients, total GSL levels in the substantia nigra were increased to 131.1% of age-matched control subjects, but did not reach statistical significance (Fig. 4l). In substantia nigra of 80s-cohort PD patients, total GSL levels were significantly increased to 165.5% of 80s-cohort control subjects (Fig. 4l).

### Reduced activity of multiple lysosomal hydrolases, including GBA, in substantia nigra from a second PD patient cohort

We also analysed hydrolase activities in a second, independent cohort of post-mortem SN tissue from healthy control subjects in their 80s (*n* = 5) and age-matched PD patients (*n* = 9) (Parkinson’s UK (PDUK) Brain Bank).

**Fig. 5:**
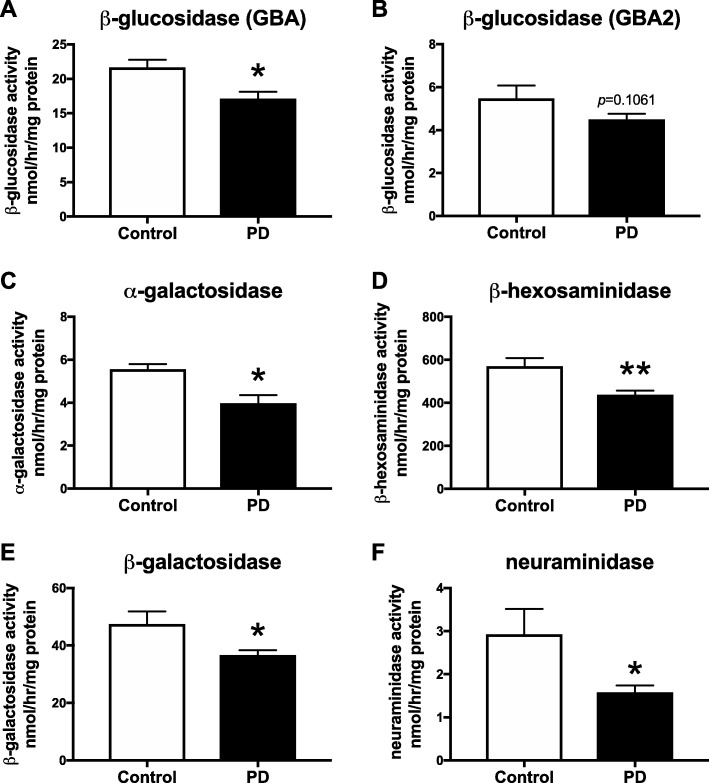
Reduced lysosomal hydrolase activities in substantia nigra from a second cohort of PD patients. Lysosomal hydrolase activities were measured using artificial 4-MU-substrates. Activity of GBA (**a**), GBA2 (**b**), α-galactosidase (**c**), β-hexosaminidase (**d**), β-galactosidase (**e**) and neuraminidase (**f**) were determined in substantia nigra from age-matched control subjects (*n* = 5) and PD patients (*n* = 9). Data were analysed using unpaired t-test (* = *p* < 0.05, ** = *p* < 0.01). Data are presented as mean ± SEM

GBA activity in substantia nigra of PD patients was significantly reduced to 79.1% of GBA activity in control subjects (Fig. 5a). Furthermore, a decrease in GBA2 activity was seen in SN of PD patients compared to control subjects (17.9% reduction, Fig. 5b). A significant reduction in α-galactosidase activity was observed in SN of PD patients compared to age-matched control subjects (28.4% reduction, Fig. 5c). Additionally, a significant reduction in β-hexosaminidase activity was detected in SN of PD patients compared to SN of control subjects (23.1% reduction, Fig. 5d). The activity of β-galactosidase in substantia nigra of PD patients was significantly reduced to 77.3% of β-galactosidase activity of age-matched control subjects (Fig. 5e). Finally, the activity of neuraminidase in substantia nigra of PD patients was significantly reduced to 54.0% of activity in SN of age-matched controls (Fig. 5f).

### Increase in glucosylceramide and decrease in gangliosides in substantia nigra from a second PD patient cohort

Additionally, we analysed GSLs in a second, independent cohort of post-mortem SN tissue from healthy control subjects (*n* = 5) and age-matched PD patients (*n* = 20) in their 80s (PDUK Brain Bank).

**Fig. 6:**
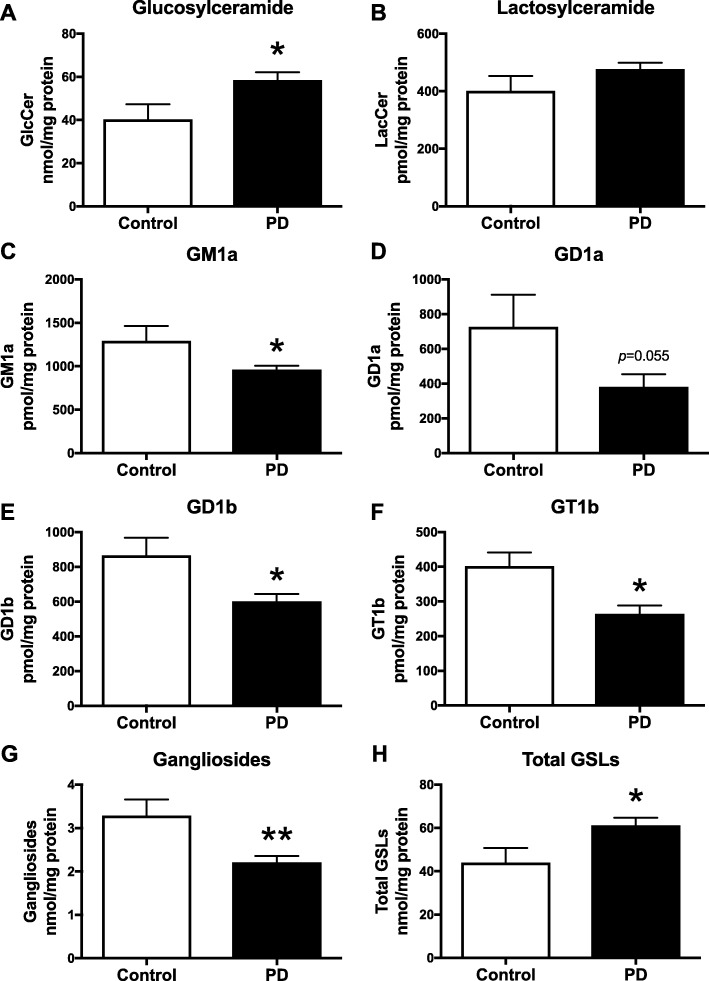
Increase in glucosylceramide and loss of gangliosides in substantia nigra from a second cohort of PD patients. Substantia nigra from control subjects (*n* = 5) and PD patients (*n* = 20) were used to determine GlcCer (**a**), LacCer (**b**) GM1a (**c**), GD1a (**d**), GD1b (**e**) and GT1b (**f**) levels with NP-HPLC (* = *p* < 0.05, unpaired t-test). **g** Comparison of total ganglioside levels (sum of GM1a, GD1a, GD1b and GT1b) in substantia nigra from control subjects and PD patients (** = *p* < 0.01, unpaired t-test). **h** Total GSL levels (sum of GlcCer + LacCer + GM1a + GD1a + GD1b + GT1b levels) in substantia nigra from control subjects and PD patients (* = *p* < 0.05, unpaired t-test). Data are presented as mean ± SEM

GlcCer levels were significantly increased in substantia nigra of PD patients compared to control subjects (45.0% increase, Fig. 6a). There were no significant changes in substantia nigra LacCer levels observed between PD patients and control subjects (Fig. 6b). A significant decrease in GM1a levels was observed in SN of PD patients compared to age-matched control subjects (25.7% reduction, Fig. 6c). A decrease in GD1a levels was found in SN of PD patients compared to controls (47.4% reduction, Fig. 6d). In addition, gangliosides GD1b and GT1b were both significantly reduced in SN of PD patients in comparison to control subjects (GD1b: 30.6% reduction; GT1b: 34.3% reduction; Fig. 6e, f). Consequently, in substantia nigra of PD patients, ganglioside levels (sum of GM1a, GD1a, GD1b and GT1b) were significantly decreased to 67.2% of age-matched control subjects (Fig. 6g). However, total GSL levels (sum of GlcCer, LacCer and gangliosides; mostly influenced by the highly abundant GlcCer) were significantly increased in substantia nigra of PD patients to 139.1% of control subjects (Fig. 6h).

### GSL biomarkers in cerebrospinal fluid from PD patients

GSLs in cerebrospinal fluid (CSF) from control subjects and PD patients were quantified as potential biomarkers. Ante-mortem CSF samples from control subjects (*n* = 15) and age-matched PD subjects (*n* = 28) were provided by the Oxford Parkinson’s Disease Centre (OPDC; Oxford, UK). The pattern of GSLs in CSF is different and more complex than the GSL pattern in the brain. Ante-mortem CSF displays a large LacCer peak and GA2 peak (o-series), with prominent peaks of GM2, GM1a, GD1a, GD1b and GT1b (a-series & b-series), but small peaks of GM3 and GD3 (precursors of a-series and b-series).

**Fig. 7:**
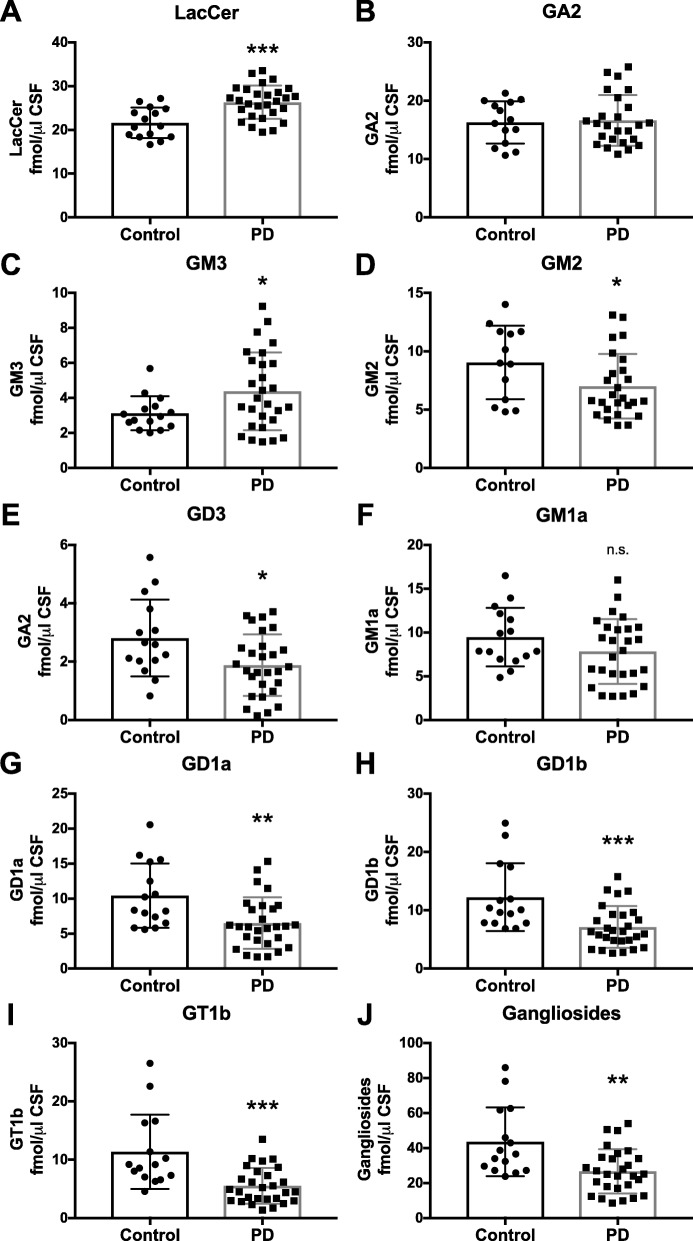
Significant increase in LacCer and GM3 levels, but significant decrease in complex ganglioside levels in CSF of PD patients. Ante-mortem CSF from control subjects (*n* = 15) and age-matched PD patients (*n* = 28) was used to determine LacCer (**a**), GA2 (**b**), GM3 (**c**), GM2 (**d**), GD3 (**e**), GM1a (**f**), GD1a (**g**), GD1b (**h**), and GT1b (**i**) levels with NP-HPLC (* = *p* < 0.05, ** = *p* < 0.01, *** = *p* < 0.001, unpaired t-test). **j** Total ganglioside levels (sum of GM1a, GD1a, GD1b and GT1b) in ante-mortem CSF from control subjects and PD patients (** = *p* < 0.01, unpaired t-test). Data are presented as mean ± SD

Owing to problems with the GlcCer digestion (inhibitory activity of CSF-derived lipids against Cerezyme), measurements of GlcCer in CSF were not possible with the NP-HPLC method. However, more complex GSLs could still be measured. LacCer levels in ante-mortem CSF of PD patients were significantly increased in comparison with age-matched control subjects (21.8% increase, Fig. 7a). There were no changes detected in GA2 levels in CSF of PD patients compared to control subjects (Fig. 7b). However, a significant increase in GM3 levels and a significant decrease in GM2 levels was found in ante-mortem CSF of PD patients compared to controls (GM3: 40.2% increase; GM2: 22.6% reduction; Fig. 7c, d). Furthermore, a significant decrease in GD3 levels was observed in CSF of PD patients compared to age-matched control subjects (33.0% reduction, Fig. 7e). We also analysed levels of more complex gangliosides of the a-series and b-series, GM1a, GD1a, GD1b and GT1b. A decrease in GM1a levels was observed in ante-mortem CSF of PD patients compared to age-matched control subjects, although it was not significant (17.4% reduction, Fig. 7f). However, a significant decrease in GD1a levels was found in CSF of PD patients compared to controls (37.6% reduction, Fig. 7g). In addition, gangliosides GD1b and GT1b were both significantly reduced in ante-mortem CSF of PD patients in comparison to control subjects (GD1b: 41.6% reduction; GT1b: 51.3% reduction; Figs. 7h, i). Consequently, in ante-mortem CSF of PD patients, complex ganglioside levels (sum of GM1a, GD1a, GD1b and GT1b) were significantly decreased to 61.4% of age-matched control subjects (Fig. 7j). Receiver Operating Characteristic (ROC) curve assessments to evaluate CSF ganglioside levels as PD biomarkers can be found in Additional file 1: Figure S4.

### GSL biomarkers in serum from PD patients and RBD patients

Finally, we analysed GSLs in serum from control subjects (*n* = 16) and age-matched PD patients (*n* = 30) (provided by OPDC, Oxford, UK) in the search for possible GSL biomarkers. The pattern of GSLs in serum is unique: GM3 (a precursor for a-series gangliosides) is the most prominent, with high levels of LacCer, Gb3 and Gb4 (globo-series), and low levels of GM2, GM1a, and GD1a (a-series).

**Fig. 8:**
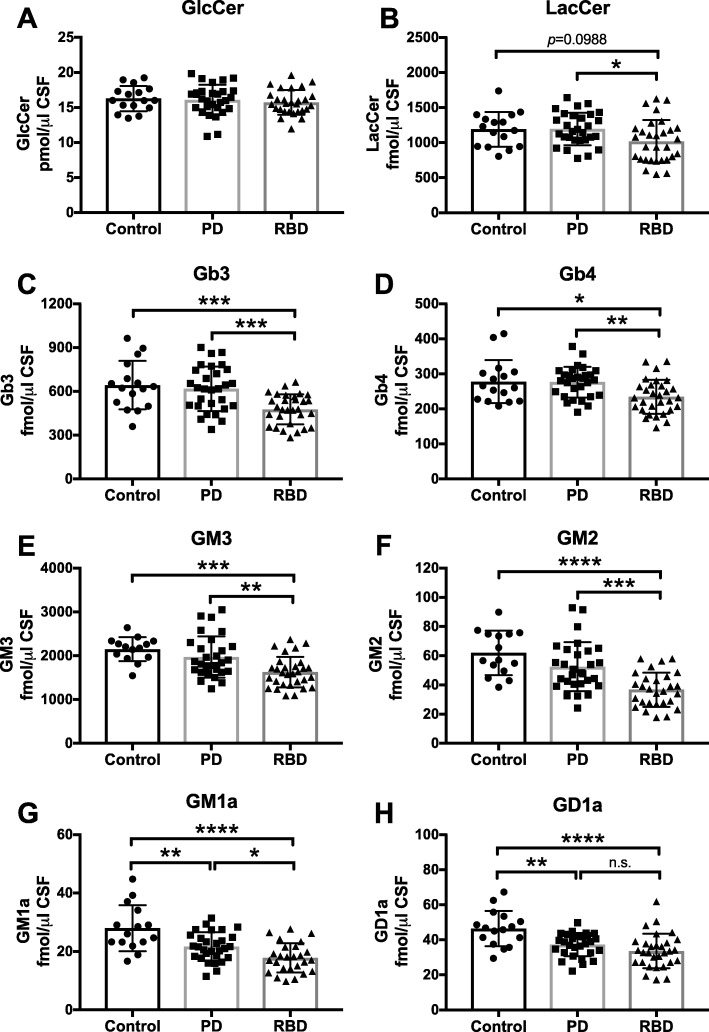
Significant reduction in GM1a and GD1a levels in serum from PD patients and significant reduction in all measured glycosphingolipids, except GlcCer, in serum from RBD patients. Levels of GlcCer (**a**), LacCer (**b**), Gb3 (**c**), Gb4 (**d**), GM3 (**e**), GM2 (**f**), GM1a (**g**) and GD1a (**h**) were determined in serum samples from control subjects (*n* = 15), PD patients (*n* = 30) and age-matched RBD patients (*n* = 30) with NP-HPLC (* = *p* < 0.05, ** = *p* < 0.01, *** = *p* < 0.001, **** = *p* < 0.0001, one-way ANOVA). Data are presented as mean ± SD

No differences in levels of GlcCer and LacCer were detected in serum of PD patients compared to age-matched control subjects (Fig. 8a, b). No changes in downstream Gb3 or Gb4 levels (globo-series) were observed in serum of PD patients in comparison with control subjects (Fig. 8c, d). No significant change in GM3 levels was found, but a trend towards a reduction in GM2 levels (a-series) was observed in serum from PD patients compared to controls (GM3: 8.3% reduction; GM2: 15.3% decrease; Fig. 8e, f). Levels of more complex gangliosides GM1a and GD1a were significantly reduced in serum from PD patients in comparison to serum from age-matched control subjects (GM1a: 22.6% reduction; GD1a: 19.8% decrease; Fig. 8g, h).

Additionally, GSLs in serum from patients at risk of developing PD (prodromal PD phase), diagnosed with REM sleep behaviour disorder (RBD, *n* = 30, provided by the OPDC), were quantified. There were no differences in GlcCer levels (Fig. 8a), but a reduction in LacCer levels was detected in serum of RBD patients in comparison to serum from PD patients (14.8% reduction, Fig. 8b). Furthermore, a significant decrease in Gb3 and Gb4 levels (globo-series) was observed in serum of RBD patients in comparison to control subjects and PD patients (Gb3: 25.8% decrease to controls, 22.8% decrease to PD; Gb4: 15.6% decrease to controls, 15.3% decrease to PD; Fig. 8c, d). A significant reduction in GM3 and GM2 levels was found in serum of RBD patients compared to serum samples from controls and PD patients (GM3: 24.4% decrease to controls, 17.6% decrease to PD; GM2: 40.7% decrease to controls, 30.0% decrease to PD; Fig. 8e, f). Levels of GM1a as well as GD1a were significantly reduced in serum from RBD patients in comparison to serum from age-matched control subjects, but less so compared to PD patients (GM1a: 36.4% decrease to controls, 17.8% decrease to PD; GD1a: 27.6% decrease to controls, 9.7% decrease to PD; Fig. 8g, h). ROC curve assessments of the utility of serum ganglioside levels as possible PD biomarkers can be found in Additional file 1: Figure S4.

## Discussion

GBA haploinsufficiency and ageing are associated with an increased risk of developing PD, and the majority of patients with sporadic PD carry at least one variant in a lysosomal storage disorder gene [52]. The present study demonstrates in two independent cohorts of post-mortem substantia nigra that PD is associated with deficiencies in several lysosomal hydrolases in addition to reduced activity of GBA and accumulation of its substrates, GlcCer and GlcSph. Moreover, we found a significant reduction in the levels of complex gangliosides in PD patient substantia nigra, and these changes to gangliosides were reflected in CSF and serum of PD patients as well as in prodromal RBD. The significance of these findings is that lysosomal enzymes and GM1a ganglioside are progressively reduced in ageing and even more significantly reduced in sporadic PD. We conclude that ageing might be a driver for disease penetration for that reason and that the pathogenesis of PD and a-synucleinopathies is intimately linked to impairments in lysosomal enzymes with concurrent glycolipid accumulation and ganglioside decline. The implications of these findings will be discussed below.

### Reduced GBA and GBA2 activity in SN with ageing and PD

Nearly 10 years ago, mutations in *GBA* were confirmed as the most common genetic risk factor for developing PD [8]. Subsequent studies demonstrated a decrease in GBA activity in brain regions from PD patients carrying a heterozygous mutation in *GBA* (GBA-PD), but also in brain regions from sporadic PD patients, not carrying a *GBA* mutation [11–13]. For example, GBA activity was significantly decreased in substantia nigra, putamen, cerebellum, and hippocampus of sporadic PD brains [11, 12]. It was also reported that GBA activity progressively declined in normal ageing in healthy controls [12], potentially explaining the fact that age is a major risk factor for developing PD. However, most published studies to date have not reliably distinguished lysosomal GBA and non-lysosomal GBA2 activities and thus analysed a mixture of β-glucosidase activities. GBA and GBA2 cleave the same substrates, thus small-molecular inhibitors are used with the aim to specifically inhibit one of these two enzymes. The commonly used compound is conduritol B epoxide (CBE), which inhibits GBA [57, 58]. However, CBE not only inhibits GBA, but also a considerable portion of GBA2 activity [14]. As the GBA2 activity in neuronal cells is relatively high, the CBE-sensitive portion of β-glucosidase activity comprises both GBA and GBA2 activities. Quantifying the GBA activity as the CBE-sensitive β-glucosidase therefore results in overestimating GBA activity [14].

Here, we have followed a different approach and carefully distinguished GBA and GBA2 activities using *N*-butyldeoxygalactonojirimycin (*N*B-DGJ), which inhibits GBA2 but does not affect GBA [14, 15]. We confirmed previously published findings [11, 12] and showed that GBA activity in substantia nigra is negatively correlated with age in control subjects and is significantly reduced in sporadic PD patients compared to age-matched controls. In addition, we confirmed a significant reduction in GBA activity in an independent cohort of SN tissue from PD patients compared to controls. However, we found for the first time that non-lysosomal GBA2 activity also has a tendency to decline in substantia nigra of control subjects with ageing, although not reaching statistical significance in our data, and is significantly reduced in PD patients compared to age-matched controls. Interestingly, it has been reported that GBA2 activity is regulated by GBA activity, but not vice versa [59]. This may explain the observed reduction in GBA2 activity in SN from PD patients with reduced GBA activity. The role of GBA2 in substantia nigra in PD needs to be further explored, but these results suggest a possible involvement of other hydrolases in PD, in addition to GBA.

### Substrate accumulation in SN of PD patients

It remains unclear whether GlcCer or GlcSph levels are elevated in PD as a direct consequence of loss of function of GBA and GBA2 activity. Analysis of putamen, cerebellum and temporal cortex samples from PD-GBA patients and sporadic PD patients showed no evidence of significant accumulation of GlcCer and GlcSph [37, 38]. On the other hand, significant GlcSph accumulation was detected in substantia nigra and hippocampus of sporadic PD patients [12]. Also, a trend for increased GlcCer levels was seen with increased PD severity [38]. In addition, galactosylsphingosine (psychosine) levels were found to be mildly elevated in the cerebral cortex in PD compared to healthy controls, but did not reach statistical significance [60].

Here, using sensitive and quantitative NP-HPLC analysis, we found a significant increase in GlcCer levels in the substantia nigra of two independent cohorts of PD patients compared to age-matched controls, as well as a significant correlation between age and GlcCer levels in substantia nigra of PD patients. Furthermore, we found a significant increase in GlcSph levels in the substantia nigra of PD patients compared to age-matched control subjects, as well as significant increases in sphingosine and sphinganine. These data support previously published findings of increased GlcSph in substantia nigra of sporadic PD patients [12], but are in contrast to other published studies reporting no changes in GlcSph or GlcCer levels in PD [37, 38]. This discrepancy between published studies is possibly due to different analytical methods and/or analysis of different brain regions, which are not necessarily expected to be affected in PD (e.g. temporal cortex), rather than substantia nigra. This could also underline the relative vulnerability of neurons in the SN in PD.

Recently, a model has been proposed in which GlcSph accumulates before GlcCer in murine GBA-PD brains [32], which agrees with our human SN data on 70s versus 80s cohorts PD subjects. Interestingly, GlcCer can be alternatively processed to GlcSph via lysosomal acid ceramidase, which can then exit the lysosome [61–63]. Thus, there may be crosstalk between GlcCer and GlcSph levels. Furthermore, it is important to note that several studies have shown that GSLs, especially GlcCer and GlcSph, interact with α-synuclein and promote the formation of assembly-state oligomeric α-synuclein species [31–34]. Moreover, there is lipidation of α-synuclein in PD and with age, associated with specific synaptic vesicles and concurrent overall loss of post-synaptic densities [64]. This indicates a possible pathological role of the observed lipid accumulation in human SN in PD via interaction with α-synuclein, and thus suggests substrate reduction therapy (SRT) as a potential treatment option. Currently, a large multi-centre clinical trial to assess the safety and efficacy of GZ402671 (Ibiglustat, Venglustat), a glucosylceramide synthase inhibitor, is ongoing with GBA-PD patients (Clinicaltrials.gov Identifier: NCT02906020, Genzyme).

### Altered lysosomal enzyme activities in PD

A recent study reported an excessive burden of putatively damaging variants of > 50 lysosomal storage disorder genes in PD [52]. This prompted us to investigate the activity of multiple lysosomal hydrolases in substantia nigra and putamen of PD patients and age-matched controls.

We found significantly reduced lysosomal α-galactosidase activity in both cohorts of substantia nigra of PD patients compared to age-matched control subjects. These data agree with a previous study showing a decrease in α-galactosidase activity and protein levels in temporal cortex in late-stage PD [65]. α-galactosidase activity was also found to be lower in dried blood spots and in leukocytes of PD patients compared to controls [66, 67]. We did not see a change in Gb3 levels, the principle GSL substrate for α-galactosidase, in substantia nigra of PD patients compared to controls. This confirms a previous study reporting that Gb3 levels were not significantly different between temporal cortex of control and PD cases [65]. Interestingly, a link between α-galactosidase and PD is supported by several lines of evidence. Firstly, pathological accumulation of α-synuclein, concomitant with disruption of autophagy-lysosome markers, has been reported in α-galactosidase A-deficient (Fabry) mouse brains [68]. Furthermore, mutations in *GLA* were found to be overrepresented in PD patients [52] and numerous Fabry patients have been diagnosed with symptoms of Parkinsonism, suggesting an increased risk of developing PD in individuals with *GLA* mutations [69–71]. However, no prodromal clinical features of parkinsonism have been identified to date in Fabry patients [72]. The physiological role of α-galactosidase in brain tissue still remains to be determined, as we did not observe Gb3 substrate accumulation and Gb3 is only expressed at very low levels in the brain. In the Fabry mouse, Gb3 is only stored in selected cells of the piriform cortex (Platt lab, unpublished data). It is therefore possible that residual enzyme activity (including α-galactosidase B activity) may be sufficient to prevent substrate accumulation, but might not be sufficient for other cellular functions.

In the present study, we also found a significant decrease in β-galactosidase and β-hexosaminidase activities in the substantia nigra of PD patients compared to age-matched control subjects. Supporting our results, reduced β-galactosidase and β-hexosaminidase activities have been reported in CSF of PD patients compared to control subjects in several studies [35, 73, 74]. Interestingly, accumulation of α-synuclein was found in brains of both β-hexosaminidase deficient Sandhoff mice and Sandhoff patients [75, 76]. Also, some patients with adult-onset GM1-gangliosidosis (deficiency in β-galactosidase) have been found to display akinetic-rigid parkinsonism [77–79]. Mutations in *GLB1* and *HEXB* were recently confirmed as LSD gene variants in PD cases [52]. These findings further support an important role for the lysosome in PD.

Finally, we report for the first time a significant decrease in neuraminidase activity in the substantia nigra of two independent cohorts of PD patients compared to age-matched controls. In addition to lysosomal degradation of gangliosides, neuraminidases can also remodel gangliosides at the plasma membrane [80, 81]. Accordingly, neuraminidases can sequentially remove sialic acid residues from GD1a, GD1b and GT1b, leading to an increase in GM1a levels. Indeed, genetic deficiency of neuraminidases 3 and 4 in mice causes a reduction in levels of GM1a [81]. We recently observed an increase in neuraminidase activity in mouse brain during normal ageing and proposed that this might reflect the observed increase in GM1a and concomitant reduction in GD1a, GD1b and GT1b levels, which could be protective for DA neurons [82]. In contrast, in human substantia nigra from PD patients, we observed a significant decrease in neuraminidase activity, which might be reflective of the observed decrease in GM1a levels. These data suggest that mice might have a compensatory, neuroprotective mechanism based on increasing neuraminidase activity and subsequent increased GM1a expression in the brain with age, which is not effective or present in the human brain. Interestingly, this seems to resemble the mechanism by which the mouse model of Tay-Sachs disease (β-hexosaminidase A deficiency, GM2 gangliosidosis) is able to escape the human disease (via degradation of GM2 to GA2 via murine neuraminidases) in contrast to the human population where neuraminidase levels are lower [83]. These findings might therefore offer one explanation as to why mice do not spontaneously develop Parkinson’s disease.

### Loss of gangliosides in human substantia nigra with ageing is more prominent in PD

In this study, we have shown that GM1a and GD1a levels of substantia nigra are negatively correlated with ageing in healthy subjects. Previous reports have indicated changes in levels of several complex gangliosides, including a progressive decline in GM1a and GD1a levels, in multiple regions of the human brain during ageing [84–86]. We demonstrate that levels of all principle brain gangliosides (GM1a, GD1a, GD1b and GT1b) are negatively correlated with ageing in substantia nigra from two independent cohorts of PD patients and that ganglioside levels are significantly reduced in substantia nigra of PD patients compared to age-matched controls. In agreement with our data, a reduction in GM1a levels in substantia nigra of PD subjects, and reductions in GM1a, GD1a, GD1b and GT1b levels in the occipital cortex of PD subjects have previously been described, using immunohistochemical staining or thin-layer chromatography [42, 45]. Interestingly, in substantia nigra of PD patients, GM1a staining with cholera toxin was diminished near α-synuclein aggregates [45]. In addition, a recent study showed reductions in GM1a, GD1a, GD1b and GT1b in the substantia nigra in a smaller cohort of PD patients using thin-layer chromatography [46].

Ganglioside metabolism and its role in PD was recently reviewed [87]. There are two important aspects of GM1a biology with regard to ageing and PD. Firstly, α-synuclein is a ganglioside-binding protein, which adopts a more stable, α-helical structure when bound to membranes, but starts to form fibrils in the absence of GM1 ganglioside [88, 89]. Secondly, GM1a is crucial for efficient signalling of the growth factor glial cell-derived neurotrophic factor (GDNF) [42]. It has been proposed that even a modest decline in GM1a ganglioside levels might inhibit this trophic support in dopaminergic neurons [87]. A significant decrease in gene expression of key biosynthetic enzymes involved in synthesis of GM1a/GD1b (*B3GALT4*) and GD1a/GT1b (*ST3GAL2*) was reported in residual neuromelanin-containing cells in the SN of PD patients compared to age-matched controls [90]. Mice deficient in the ability to synthesise a-series gangliosides (genetic deletion of *B4GALNT1*, encoding GM2 synthase), specifically GM1a, develop parkinsonism, including the loss of TH-positive cells, lower striatal dopamine levels, an accumulation of α-synuclein aggregates and impaired motor function [39]. GM2 synthase deficiency in humans results in severe spastic paraplegia [91], which may reflect a more central role for gangliosides in myelinated neurons in humans compared to mice. Intriguingly, treatment with exogenous GM1a has been reported to be beneficial in several preclinical models of PD [92–96] and in PD patients [97–99]. Additionally, deletion of GD3 synthase, which leads to an increase in GM1a ganglioside, was neuroprotective in a preclinical PD model [100].

In conclusion, the observed depletion of GM1a in human substantia nigra during normal ageing, and to a greater extent in PD, might contribute to the development of PD, rather than accumulation of the protein α-synuclein.

### Biomarkers for PD

There is an urgent need to find biomarkers for PD. Several studies have demonstrated altered activities of various lysosomal hydrolases in CSF from PD patients. For example, decreased GBA, β-hexosaminidase and β-galactosidase activities have been reported in CSF of PD-GBA patients, but also sporadic PD patients [35, 73, 74]. However, no studies have been published regarding GSLs in CSF of PD patients. We found significant changes in levels of LacCer, and most gangliosides of the a-series and b-series in PD patient CSF compared to age-matched controls. Reduced levels of more complex gangliosides GM1a, GD1a, GD1b and GT1b were also detected, in agreement with our results obtained with substantia nigra from PD patients. Consequently, alterations in ganglioside levels in ante-mortem CSF might serve as biomarkers for PD.

Plasma or serum of sporadic PD patients contains increased levels of ceramide, monohexosylceramides (GlcCer and GalCer), LacCer and GM3 compared to controls [36, 101]. Comparing sporadic PD patients with GBA-associated PD patients, serum of GBA-PD patients displayed higher levels of monohexosylceramides (GlcCer/GalCer), GlcSph and LacCer [102]. Here, we report similar levels of GlcCer, LacCer and globo-series gangliosides Gb3 and Gb4 in serum from PD patients compared to age-matched control subjects. However, we found changes in a-series gangliosides, namely a trend towards decreased GM3 and GM2 levels and significantly decreased GM1a and GD1a levels, in the serum of PD patients compared to controls.

Further studies with higher patient numbers and more refined methods are needed in the future. Nevertheless, the observed significant reduction in levels of more complex gangliosides GM1a and GD1a is in accordance with our results obtained with human substantia nigra and ante-mortem CSF from PD patients.

Finally, GSL levels in serum from patients diagnosed with REM sleep behaviour disorder (RBD), who are at significant risk of developing PD, were analysed. We found no changes in GlcCer levels in the serum from RBD patients compared to serum from control subjects or PD patients. However, we report for the first time significantly reduced levels of LacCer, globo-series gangliosides, and a-series gangliosides in the serum of RBD patients. It is interesting that RBD patients have lower serum levels of the gangliosides GM1a and GD1a than PD patients. One hypothesis is that fundamental changes in GSL levels in RBD patients might be intrinsic to the disease itself and might predispose these patients to develop PD over time. Supporting this notion, single-nucleotide polymorphisms in the *SCARB2* gene, encoding the lysosomal integral membrane protein 2 (LIMP-2), an important receptor for trafficking GBA to the lysosome, were significantly associated with RBD [103].

In summary, reduced levels of gangliosides, e.g. GM1a and GD1a, in serum and CSF might have the potential to be adjunctive PD biomarkers for monitoring disease progression, for stratifying patients for clinical trials and for determining responses to new therapies.

## Conclusions

In conclusion, we have shown here for the first time that both lysosomal GBA and non-lysosomal GBA2 activities are negatively correlated with ageing in the substantia nigra of control subjects and are significantly reduced in sporadic PD patients compared to age-matched controls. In addition, we have confirmed the results of Rocha and co-workers [12]*,* and found significant substrate accumulation (GlcCer and GlcSph) in SN in PD. Furthermore, we showed that multiple lysosomal enzymes have significantly reduced activities in the substantia nigra of PD patients compared to controls. Finally, this is the first extensive, quantitative study of gangliosides in ageing and PD showing a negative correlation of more-complex gangliosides, i.e. GM1a, with ageing. Importantly, levels of these gangliosides were reduced to a greater extent in substantia nigra of PD patients compared to age-matched controls. All these results were confirmed in two independent cohorts of PD patients, one from the US and one from the UK. Finally, this is the first report of significant reductions in complex gangliosides in CSF and serum of PD patients as well as prodromal RBD patients compared to control subjects.

Taken together, these findings demonstrate that not only a reduction in GBA activity may lower the threshold for developing PD, but substrate accumulation, reduced activities of other lysosomal hydrolases and reduced levels of neurotrophic complex gangliosides may also be detrimental for SN neurons and increase the risk of developing PD. Importantly, therapies improving lysosomal function and modulating activities of lysosomal enzymes and levels of GSLs could exert beneficial effects in PD.

## Supplementary information

**Additional file 1: Figure S1.** No change in Gb3 levels in substantia nigra of PD patients. (A) Substantia nigra from control subjects (*n* = 20) and PD patients (*n* = 18) were used to determine Gb3 levels with NP-HPLC. Data were analysed using Pearson correlation analysis. (B) Comparison of Gb3 levels in 70s-cohorts and 80s-cohorts of control subjects and PD patients (*n* = 8–10 per cohort, 2-way ANOVA). Bar graphs are presented as mean ± SEM. **Figure S2.** HPLC traces of glucosylceramide and gangliosides GM1a, GD1a, GD1b and GT1b extracted from substantia nigra of control subjects and PD patients*.* Exemplary NP-HPLC traces of (A) GlcCer and (B) gangliosides of 80s-cohort control subjects are shown in grey (*n* = 3) and 80s-cohort PD patients are shown in red (*n* = 3). **Figure S3.** Substantia nigra cholesterol levels are unchanged with normal ageing or in PD. Comparison of total cholesterol levels in substantia nigra from control subjects and PD patients of both 70s-cohorts and 80s-cohorts (*n* = 5 per cohort, 2-way ANOVA). Cholesterol levels were analysed with Amplex Red kit. Data are presented as mean ± SEM. **Figure S4.** Receiver Operating Characteristic (ROC) curve assessment of the utility of ganglioside levels in serum and CSF of PD patients as possible biomarkers. Comparison of PD patients (*n* = 30) and age-matched controls (*n* = 15) using GM1a (A), GD1a (B), GD1b (C), GT1b (D) and total ganglioside (E) levels in CSF and GM1a (F) and GD1a (G) levels in serum as biomarkers. The dashed line represents the line of no discrimination. AUC = Area under curve.

**Additional file 2.** Data of individual patients.

**Additional file 3.** Statistics.

## Data Availability

The data generated during the current study are available from the corresponding author on reasonable request.

## Abbreviations

2-AA: Anthranilic acid
4-MU: 4-Methylumbelliferone
CBE: Conduritol B epoxide
CSF: Cerebrospinal fluid
GBA: Glucocerebrosidase or acid β-glucosidase
GBA2: Non-lysosomal β-glucosidase 2
GD: Gaucher disease
GlcCer: Glucosylceramide
GlcSph: Glucosylsphingosine
GSL: Glycosphingolipid
HBTRC: Harvard Brain Tissue Resource Centre
HPLC: High-performance liquid chromatography
LacCer: Lactosylceramide
LSDs: Lysosomal storage disorders
*N*B-DGJ: *N*-butyldeoxygalactonojirimycin
NP-HPLC: Normal-phase high-performance liquid chromatography
OBB: Oxford Brain Bank
OPDC: Oxford Parkinson’s Disease Centre
PD: Parkinson’s disease
PDUK: Parkinson’s UK charity
RBD: Rapid eye movement sleep behaviour disorder
rEGCase: Recombinant endoglycoceramidase, recombinant ceramide glycanase
REM: Rapid eye movement
ROC: Receiver Operating Characteristic
RP-HPLC: Reverse-phase high-performance liquid chromatography
RT: Room temperature
SN: Substantia nigra
Sph: Sphingosine
SphA: Sphinganine
