# Correction to: Reduced sphingolipid hydrolase activities, substrate accumulation and ganglioside decline in Parkinson’s disease

**DOI:** 10.1186/s13024-019-0355-z

**Published:** 2020-01-15

**Authors:** Mylene Huebecker, Elizabeth B. Moloney, Aarnoud C. van der Spoel, David A. Priestman, Ole Isacson, Penelope J. Hallett, Frances M. Platt

**Affiliations:** 1 Department of Pharmacology, University of Oxford, Oxford, OX1 3QT UK; 2 Neuroregeneration Institute, McLean Hospital / Harvard Medical School, Belmont, MA 02478 USA; 3 Departments of Pediatrics and Biochemistry & Molecular Biology, Atlantic Research Centre, Dalhousie University, Halifax, NS B3H 4R2 Canada

**Correction to: Mol Neurodegener**

**https://doi.org/10.1186/s13024-019-0339-z**

The original article [1] contains an error in the y-axes of Fig. 8’s sub-figures whereby ‘CSF’ is mistakenly mentioned instead of ‘serum’.

The correct version of Fig. 8 can be viewed ahead and should be considered in place of the original Fig. 8.

**Fig. 8:**
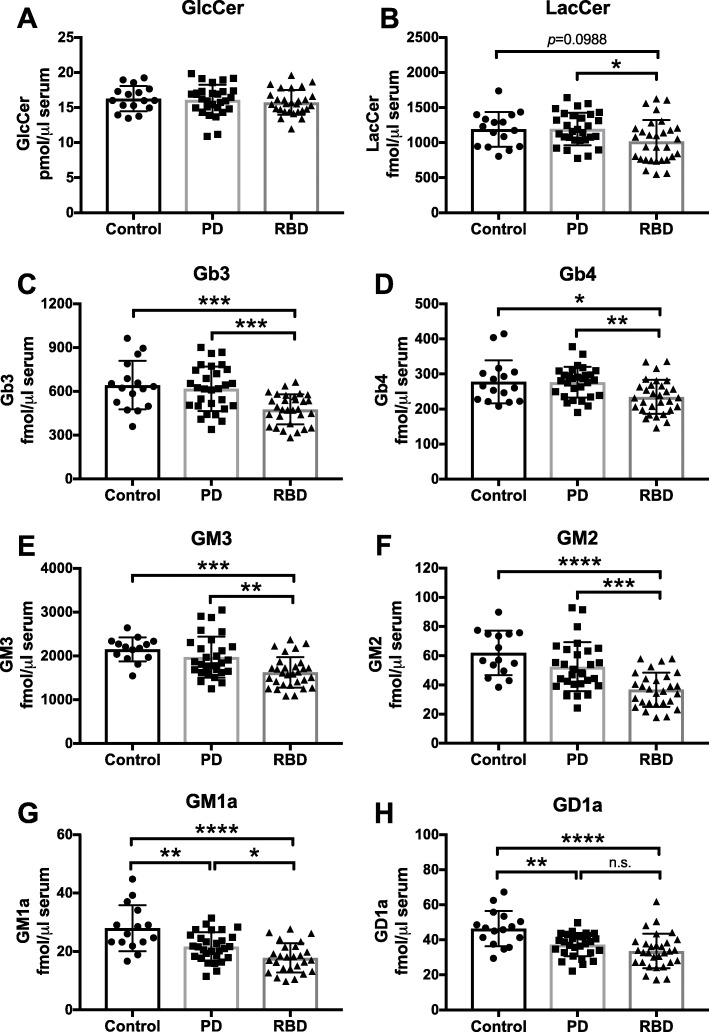
Significant reduction in GM1a and GD1a levels in serum from PD patients and significant reduction in all measured glycosphingolipids, except GlcCer, in serum from RBD patients. Levels of GlcCer (**a**), LacCer (**b**), Gb3 (**c**), Gb4 (**d**), GM3 (**e**), GM2 (**f**), GM1a (**g**) and GD1a (**h**) were determined in serum samples from control subjects (*n* = 15), PD patients (*n* = 30) and age-matched RBD patients (*n* = 30) with NP-HPLC (* = *p* < 0.05, ** = *p* < 0.01, *** = *p* < 0.001, **** = *p* < 0.0001, one-way ANOVA). Data are presented as mean ± SD
