# Supplementary figures and images for: Reduced sphingolipid hydrolase activities, substrate accumulation and ganglioside decline in Parkinson’s disease
Source: Mol Neurodegener. 2019 Nov 8;14:40. doi: 10.1186/s13024-019-0339-z (PMC6842240; doi:10.1186/s13024-019-0339-z)

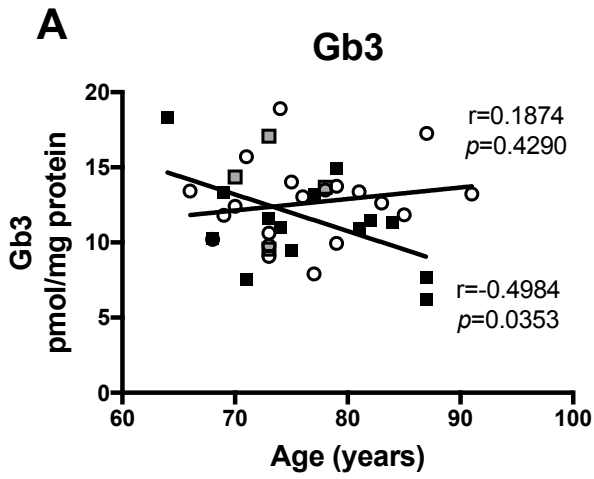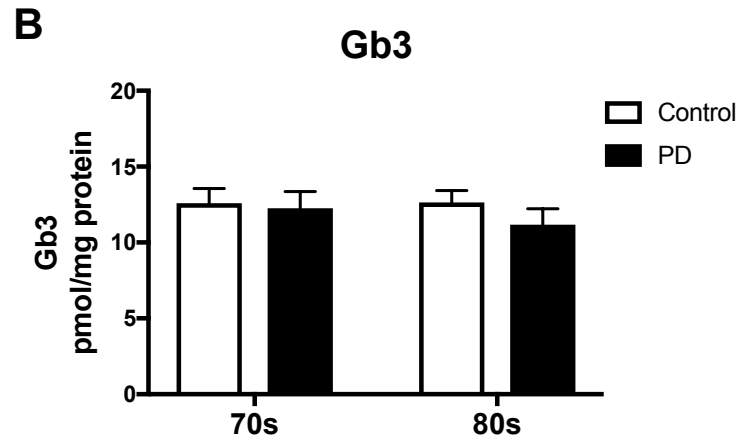

**A**
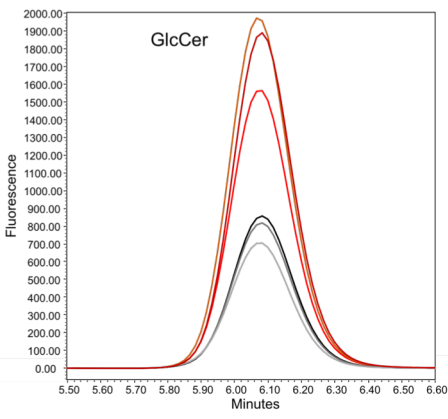
**B**
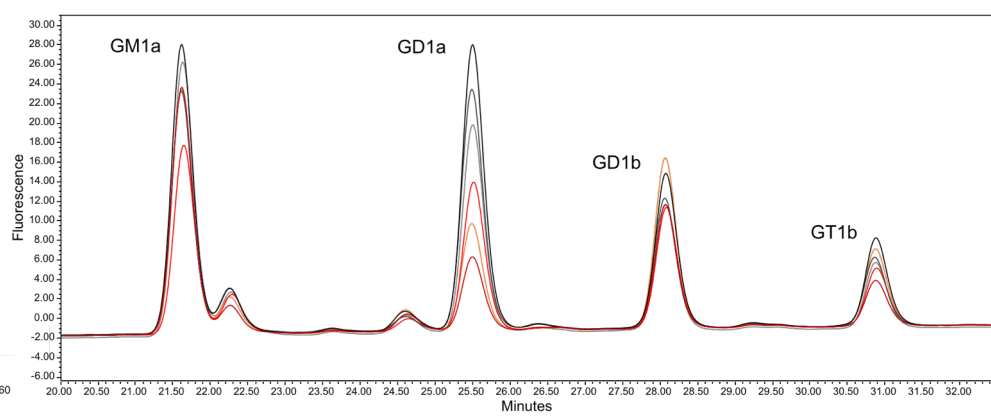

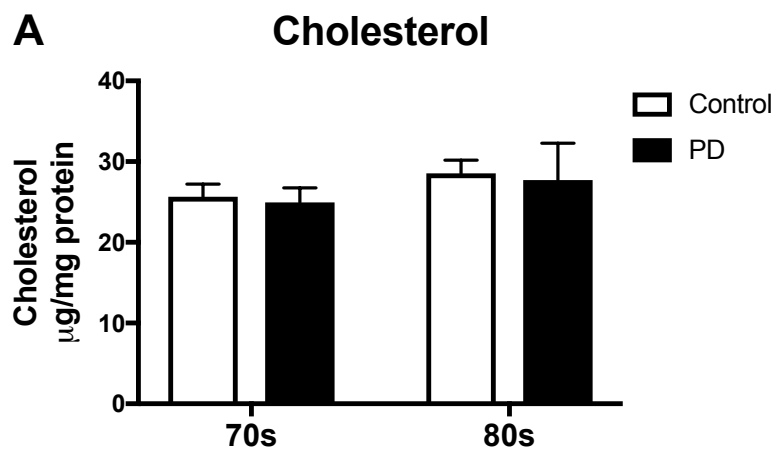

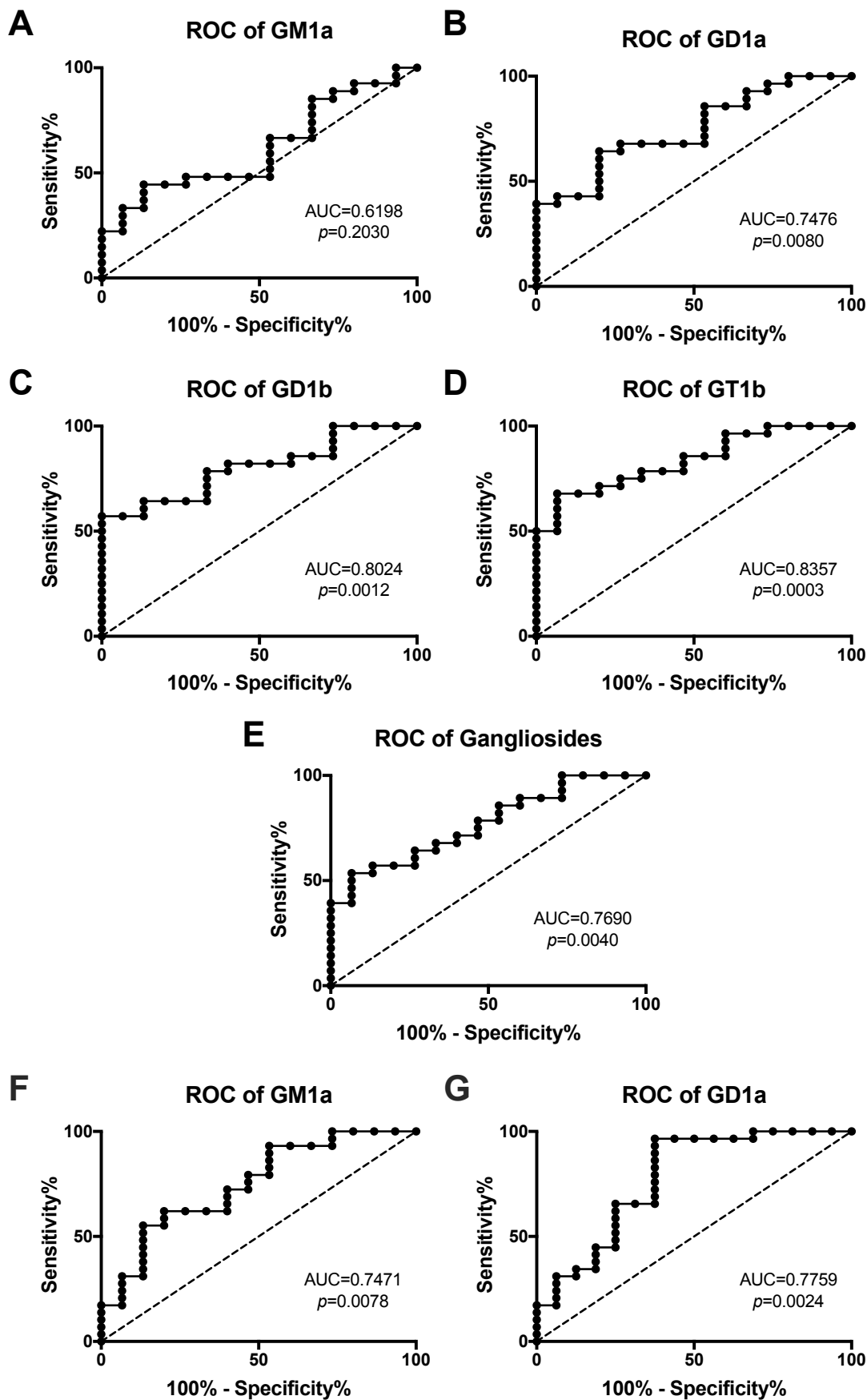

Supplement: Supplementary file 1 — Additional file 1: Figure S1. No change in Gb3 levels in substantia nigra of PD patients. (A) Substantia nigra from control subjects (n = 20) and PD patients (n = 18) were used to determine Gb3 levels with NP-HPLC. Data were analysed using Pearson correlation analysis. (B) Comparison of Gb3 levels in 70s-cohorts and 80s-cohorts of control subjects and PD patients (n = 8–10 per cohort, 2-way ANOVA). Bar graphs are presented as mean ± SEM. Figure S2. HPLC traces of glucosylceramide and gangliosides GM1a, GD1a, GD1b and GT1b extracted from substantia nigra of control subjects and PD patients. Exemplary NP-HPLC traces of (A) GlcCer and (B) gangliosides of 80s-cohort control subjects are shown in grey (n = 3) and 80s-cohort PD patients are shown in red (n = 3). Figure S3. Substantia nigra cholesterol levels are unchanged with normal ageing or in PD. Comparison of total cholesterol levels in substantia nigra from control subjects and PD patients of both 70s-cohorts and 80s-cohorts (n = 5 per cohort, 2-way ANOVA). Cholesterol levels were analysed with Amplex Red kit. Data are presented as mean ± SEM. Figure S4. Receiver Operating Characteristic (ROC) curve assessment of the utility of ganglioside levels in serum and CSF of PD patients as possible biomarkers. Comparison of PD patients (n = 30) and age-matched controls (n = 15) using GM1a (A), GD1a (B), GD1b (C), GT1b (D) and total ganglioside (E) levels in CSF and GM1a (F) and GD1a (G) levels in serum as biomarkers. The dashed line represents the line of no discrimination. AUC = Area under curve. [file 13024_2019_339_MOESM1_ESM.pdf]
